# Matrix Effects of the Hydroethanolic Extract of Calyces of *Physalis peruviana* L. on Rutin Pharmacokinetics in Wistar Rats Using Population Modeling

**DOI:** 10.3390/pharmaceutics13040535

**Published:** 2021-04-12

**Authors:** Gina Paola Domínguez Moré, María Isabel Cardona, Paula Michelle Sepúlveda, Sandra Milena Echeverry, Cláudia Maria Oliveira Simões, Diana Marcela Aragón

**Affiliations:** 1 Departamento de Farmacia, Facultad de Ciencias, Universidad Nacional de Colombia, Bogotá 11011, Colombia; gpdominguezm@unal.edu.co (G.P.D.M.); micardonap@unal.edu.co (M.I.C.); pmsepulvedar@unal.edu.co (P.M.S.); smecheverryg@unal.edu.co (S.M.E.); 2 Centro de Servicios Farmacéuticos y Monitoreo de Fármacos, Facultad de Química y Farmacia, Universidad del Atlántico, Puerto Colombia 081001, Colombia; 3 Programa de Pós-Graduação em Farmácia, Universidade Federal de Santa Catarina—UFSC, Florianópolis 88040-970, Brazil; claudia.simoes@ufsc.br

**Keywords:** rutin, pharmacokinetics, population pharmacokinetic modeling, extract, *Physalis peruviana*

## Abstract

Rutin is the rutinose conjugate of quercetin. It presents several biological activities and is the major flavonoid in the hydroalcoholic extract of the calyces of *Physalis peruviana* L. It also shows hypoglycemic activity after oral administration. The aim of this work was to study the matrix effects of the extract from *P. peruviana* calyces on the pharmacokinetics of rutin and its metabolites in Wistar rats, using non-compartmental and population pharmacokinetic analyses. A pharmacokinetic study was performed after intravenous and oral administration of different doses of pure rutin and the extract. In the non-compartmental analysis, it was found that rutin from the extract exhibited higher distribution and clearance, as well as an 11-fold increase in the bioavailability of its active metabolites. A population pharmacokinetic model was also carried out with two compartments, double absorption and linear elimination, in which the extract and the doses were the covariates involved. This model correctly described the differences observed between rutin as a pure compound and rutin from the extract, including the dose dependency.

## 1. Introduction

Rutin (quercetin-3-*O*-rutinoside) is a glycosylated flavonoid that is widely distributed in nature and presents several biological activities, including antidiabetic and anti-inflammatory effects [1,2,3,4,5]. This compound is the major flavonoid in a hydroalcoholic extract of calyces of *Physalis peruviana* L., a tropical plant of the Solanaceae family used in South American folk medicine [6,7]. Previously research conducted by our group demonstrated the oral hypoglycemic activity of this extract in rodents, and efforts have been made to describe its pharmacokinetic (PK) and pharmacodynamic profiles in order to design a new botanical preparation [8,9,10,11].

**Figure 1:**
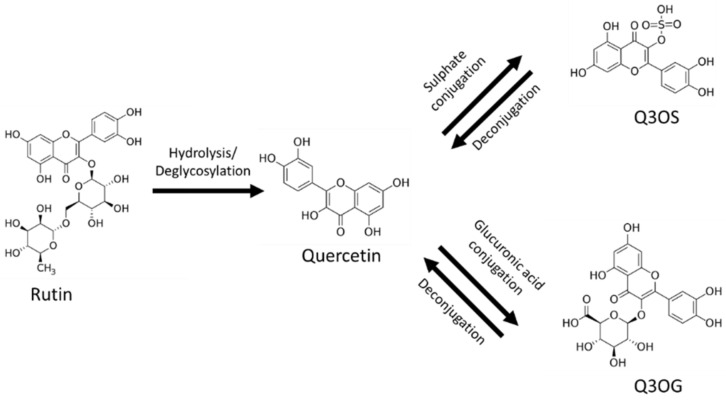
Rutin, quercetin, quercetin-3-*O*-sulfate (Q3OS), quercetin-3-*O*-glucuronide (Q3OG).

The main barrier to the use of rutin as a therapeutic drug is its poor oral bioavailability, placing it in class IV of the biopharmaceutical classification system (low solubility and low permeability) [1,9,12]. After oral administration, most of the rutin is deglycosylated in the gastrointestinal tract by reactions mainly catalyzed by the enzymes *β*-glucosidase and *α*-rhamnosidase from gut flora; these reactions produce quercetin, which, in turn, is conjugated with glucuronic acid or sulfate in the enterocytes (Figure 1). For this reason, quercetin-3-*O*-glucuronide (Q3OG) and quercetin-3-*O*-sulfate (Q3OS) are the principal compounds detected in plasma after oral administration of rutin and are used as traceable metabolites in PK studies [13,14,15]. Once formed, quercetin conjugates can remain inside the enterocytes, entering the blood circulation or returning to the intestinal lumen via P-gp (P-glycoprotein) transport (efflux). A portion of the effluxed fraction can be reabsorbed in the small intestine or the colon, producing a double-peak phenomenon in the plasma concentration–time curve [16,17,18].

In recent years, researchers worldwide have shown increased interest in the pharmacokinetics of plant extracts and have analyzed the plasma concentrations of marker compounds, i.e., molecules that contribute to the pharmacological effects of the extracts or act as analytical markers [19]. Some studies have concluded that the complex mixture of compounds in a plant extract can affect the PK of pure markers by modifying their solubility and permeability characteristics or through PK interactions [20,21,22,23,24,25,26]. Such matrix effects have been described by several authors for rutin and its conjugated metabolites, and those studies have shown that the solubility, permeability, efflux, and bioavailability of rutin or its conjugates increase or decrease depending on the matrix composition [21,27,28,29,30,31].

The hydroalcoholic extract of calyces of *P. peruviana* contains another flavonoid in addition to rutin: nicotiflorin (kaempferol-3-*O*-rutinoside) [11,32]. Additional compounds reported for these calyces are sucrose esters [33], phytoprostanes, and withanolides [34]. A recent investigation conducted by our group found that the matrix of this extract increases rutin solubility, permeability, and metabolism in the enterocytes when compared with the pure compound [9]. Thus, we hypothesized that the matrix also affects the PK processes and the bioavailability of rutin and its conjugates.

Population pharmacokinetic (popPK) is a nonlinear mixed-effects modeling approach used to gain a better understanding of the PK behavior of compounds, including the source of variability, at the preclinical and clinical stages of drug discovery and in the regular clinical setting. This kind of modeling also allows the time course of drug and the PK parameters to be predicted for individuals or populational groups with special conditions and enables situations of clinical or technical relevance to be simulated [35,36]. PopPK analysis has also been used to explore drug–drug interactions [37] and, in rare cases, for the PK description of botanical drugs with reliable results [38]. In this research, we use preclinical popPK modeling to better describe the effects of the multicomponent of the extract on rutin PK and to obtain useful information for the design of a new, safe, and effective botanical preparation for human use.

Thus, the aim of this work was to study the matrix effects of the hydroalcoholic extract of *P. peruviana* calyces on rutin PK in rats, using non-compartmental and popPK analyses. We identified that the PK parameters of rutin are different between pure compound and extract and presented a new application for popPK in the description of such changes.

## 2. Materials and Methods

### 2.1. Chemicals and Materials

UHPLC-grade methanol, acetonitrile, and formic acid were purchased from Merck (Darmstadt, Germany). The standard compounds rutin trihydrate (89.0% of anhydrous rutin), quercetin (95.0%), quercetin-3-*O*-glucuronide (Q3OG, 98.1%), chrysin (99%), the enzyme mixes β-glucuronidase/arylsulfatase, and dimethyl sulfoxide (DMSO) were obtained from Sigma-Aldrich (Deisenhofen, Germany). Water was purified using a Milli-Q system from Millipore (Bedford, MA, USA). All other chemicals were of analytical reagent grade.

### 2.2. Preparation of Hydroalcoholic Extract from Calyces of P. peruviana

The extract was prepared according to previous publications of our group [9,39]. Briefly, calyces collected in Granada, Cundinamarca-Colombia (identified under record no. COL 512200, Colombian National Herbarium) were dried, pulverized, percolated with 70% ethanol (1:15 *m*/*v*) for 72 h, evaporated under reduced pressure, and then lyophilized. The rutin content in the extract was confirmed by the validation method of Cardona [39].

### 2.3. Animals

The PK studies of rutin, both pure and from the extract of calyces of *P. peruviana*, were carried out using 50 male Wistar rats (7–11 weeks old and 250–300 g) obtained from the animal facility of the Department of Pharmacy of the Universidad Nacional de Colombia. The animals were kept under constant temperature conditions (22 ± 1 °C), with light/dark cycles of 12 h. They were provided with water and food ad libitum and fasted for 12 h before the assays. This protocol was approved by the ethics committee of the science faculty (Act 06, 2015, project 40831, 22 June 2015).

### 2.4. Pharmacokinetics Studies

Animals were randomly assigned to groups (*n* = 5) that received the interventions indicated in Table 1. For intravenous (i.v.) administration, the extract was prepared in sodium chloride solution (0.9% NaCl), vortexed, and sonicated 3–4 times for 15 min. Rutin was first dissolved in DMSO and then diluted in 0.9% NaCl. The clear solutions were administrated via the lateral tail vein of the rats. For the oral route (p.o.) experiments, the extract and rutin were suspended in water close to the time for oral gavage dosing. The highest i.v. dose of pure rutin (2.9 mg/kg) resulted in the maximum amount of flavonoid dissolving in the dosage volume (1 mL/250 g of body weight), while keeping DMSO <0.1%. Rutin doses below 1.45 (i.v.) or 75 (p.o.) mg/kg could not show enough quantifiable analytical signals in blood plasma. For the extract, the doses were close to the active ones, according to previous studies by our group [8,10].

Samples of the animals’ blood (approximately 250 µL) were taken by puncture in the lateral tail vein and collected in heparinized tubes at times 0, 0.05, 0.166, 0.333, 0.5, 0.75, 1, 1.5, 2, 3, 4, 6, 8, and 12 h for the i.v. experiments and at times 0, 0.083, 0.25, 0.30, 0.75, 1, 2, 3, 4, 6, 8, 10, 12, and 24 h for the p.o. experiments. Blood plasma aliquots were separated in new tubes by centrifugation at 3200× *g* and at 4 °C for 15 min, immediately acidified at pH 4 with 0.5 μL of formic acid (42.5%), and frozen at −80 °C until analyses.

### 2.5. Bioanalyses

Blood plasma aliquots (50 µL) were added to a chrysin standard solution (internal standard (IS)) at a final concentration of 1000 ng/mL. Proteins were precipitated with methanol (1:2 v:v), and clear supernatants were obtained by centrifugation at 1200× *g* and at 4 °C for 15 min; 6 µL of each aliquot was used for instrumental analyses. Another 50 µL of blood plasma aliquots with the IS were treated with β-glucuronidase/arylsulfatase (120 Fishman units) in order to indirectly quantify the Q3OG and quercetin-3-*O*-sulfate (Q3OS) present in these samples by conversion into the parent quercetin (deconjugation). The deconjugation reaction was promoted by incubating the samples with the enzymes at pH 5.5 and 37 °C for 30 min. Reactions with the Q3OG standard (10,000 ng/mL) and blank blood plasma were included in each analysis set as positive and negative controls, respectively. After the reaction, proteins were precipitated, as indicated above.

Rutin and quercetin quantification was carried out by UHPLC-UV using a Chromaster RS chromatograph (Hitachi, Tokyo, Japan) and a Kinetex^®^ EVO C18 column (2.6 µm, 100 × 2.1 mm; Phenomenex, Torrance, CA, USA) with an oven setting at 30 °C and a UV detector at 260 nm. The mobile phase consisted of 0.1% formic acid in water (A) and in acetonitrile (B), flowing at 0.5 mL/min in a gradient from 75% A to 65% A up to 9.3 min and 5 min of re-equilibration. The method was validated according to FDA guidelines [40].

### 2.6. Pharmacokinetic Analysis

#### 2.6.1. Non-Compartmental Analysis

The PK parameters of rutin and quercetin (produced by deconjugation of Q3OG and Q3OS) were first determined by non-compartmental analysis (NCA) using the software program Pkanalix 2019R1 (Lixoft^®^, Paris, France), set to linear trapezoidal integral mode. The area under the curve to infinity (AUC_0-INF_), clearance (Cl), volume of distribution at the steady state (Vdss), volume of distribution associated with the terminal phase (Vdz), first-order rate constant of elimination (k), terminal half-life (t_1/2_), mean residence time (MRT), maximum observed concentration (Cmax), and time of maximum observed concentration (tmax) were determined for the analytes detected for each intervention.

The relative bioavailability (F_rel_) of the extract to pure rutin was calculated by the equation

(1) $$F_{rel}=\frac{AUC_{0-INF\;extract}}{{AUC}_{0-INF\;rutin}}\times \frac{D_{rutin}}{D_{extract}},$$

where *D* is the dose and *extract* refers to rutin in the extract.

#### 2.6.2. Population Pharmacokinetic Modeling

Population pharmacokinetic (PopPK) modeling for rutin and quercetin (produced by deconjugation of Q3OG and Q3OS) was performed using the software program Monolix 2019R1 (Lixoft^®^, Paris, France). One- and two-compartment structural models with first-order elimination were evaluated, and double first-order absorption was included for the oral dosing experiments. Analyses of the categorical covariates (pure rutin, extract, and doses) were performed, and those that were significant were added to the model (*p* < 0.05 for Pearson’s and Wald tests with stochastic approximation). Correlations between random effects of the parameters were also tested and added to the model, where appropriate (*p* < 0.05 for *t*-test). The final model, including the model of the residual error, was selected based on the observation of individual fit plots, Akaike’s information criteria (AIC) values, visual assessment of the observation-versus-prediction plot, the precision of the estimated parameters, and evaluation of the distribution of residuals [41]. The model was internally validated by a visual predictive check (VPC) with 1000 simulated data.

The general model equation for the PK parameters was

(2) $$P_{i}=P_{pop}+\beta_{covariate\;n}+\eta_{\left(i,p\right)},$$

where *P_i_* is the individual parameter, *P_pop_* is the population parameter, *β* is a parameter representing the variability due to the significant covariate *n*, and *η_(i,p)_* is the individual deviation from *P_pop_*. According to the probability distribution of individual parameters, *P* in the equation could be expressed as log*P* or logit*P* (log*P*/((1 − *P*)).

### 2.7. Statistical Analyses

Data are represented as the mean ± standard deviation (SD). Group comparison was performed by simple one-way ANOVA followed by Tukey’s test (HSD) or the Mann–Whitney test. Comparisons between two groups were carried out using Student’s *t*-test or the Wilcoxon test. All statistical tests were performed using the software program Statgraphics Centurion XVI v.16.1.02, considering a significance level of α = 0.05.

## 3. Results and Discussion

### 3.1. Rutin Content in the Extract of Calyces of P. peruviana

The rutin content in the extract of calyces of *P. peruviana* was 14.80 ± 0.3 µg/mg. A representative chromatogram of the extract is presented in Figure S1.

### 3.2. Bioanalytical Method Validation

The UHPLC-UV method used for rutin and quercetin quantification in the blood plasma samples was selective and specific for these analytes and for the IS (Figure S2) and met all the criteria of the FDA guidelines [40]. Linearity was described for the equations *y* = 0.00042*x* + 0.00910 (*r*^2^ 0.9996) and *y* = 0.00056 − 0.00767 (*r*^2^ 0.99980) for rutin and quercetin, respectively, at the concentration range from 100 to 10,000 ng/mL with a 1/x^2^ weighting factor. Accuracy and precision were ≥85.6% (as recovery) and ≤7.2% (as variation coefficient), respectively, for both analytes, and these parameters were not affected by dilutions of over-concentrated samples up to 20 times the upper limit of quantification (recovery ≥ 86.3%). Rutin and quercetin remained stable in plasma at acidic pH after a short period of refrigeration (2 h, 4 °C), a long period of freezing (30 d, −80 °C), and 3 freeze/thaw cycles (recovery ≥ 87.8%).

### 3.3. Non-Compartmental Pharmacokinetic Analysis (PK-NCA)

#### 3.3.1. Rutin PK-NCA from Intravenous Administration

**Figure 2:**
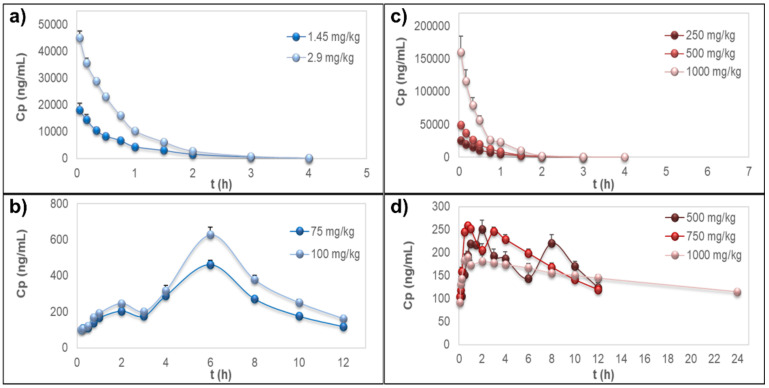
Plasma concentration–time curves. (**a**) Rutin after intravenous administration of the pure compound. (**b**) Quercetin representing the metabolites quercetin-3-*O*-glucuronide (Q3OG) and quercetin-3-*O*-sulfate (Q3OS) after oral administration of pure rutin. (**c**) Rutin after intravenous administration of the extract of calyces of *Physalis peruviana*. (**d**) Quercetin representing the metabolites Q3OG and Q3OS after oral administration of the extract of calyces of *P. peruviana*. Data are expressed as the mean ± standard error of the mean (*n* = 5).

The time–course curves of rutin plasma concentrations, after i.v. administration of the pure compound or the extract, showed rapid elimination of the flavonoid without detectable signals 4 h post-dosing (Figure 2a,b); however, the extrapolated AUC values were <20%. PK parameters from the NCA of rutin are detailed in Table 2. Short MRT values were observed for both sources (MRT ≤ 1.08 ± 0.176 h). In these experiments, no free quercetin was detected in the samples for any treatment, dose, or sampling time.

The AUC_0-INF_ within groups was proportional to the dose, which is a characteristic of linear PK [42]. The matrix effects of the extract on rutin distribution and elimination were expressed as an increase in Vdss, Vdz, and Cl up to 2.6, 2.2, and 3.0 times, respectively, which also produced an increase in k up to 1.8 times and a decrease in the MRT and t_1/2_ up to 50%, always comparing with the pure compound (Table 2).

According to the literature, rutin binds to serum albumin but with a lower affinity than simpler polyphenols [43,44,45,46]. Since less protein binding of the drug means a freer fraction and more volume of distribution, the increase observed in Vdss and Vdz of rutin may be due to the saturation of blood plasma proteins or to displacement of rutin by other components of the extract with higher affinity. The same event would lead to an increase in Cl, since the greater free fraction produced would be liable to rapid excretion by non-saturable mechanisms such as glomerular filtration [47,48]. The increase in the volume of distribution by the matrix of an extract has been reported for other polyphenols, such as osthol [26].

#### 3.3.2. Quercetin Conjugates PK-NCA after Oral Administration

Pure rutin or oral administration of the extract to the rats did not allow measurable plasma concentrations of rutin or free quercetin but only of quercetin after deconjugation of Q3OG and Q3OS, which showed a double-peak at the plasma concentration–time curve (Figure 2c,d). Considering that Q3OG is also a pharmacologically active polyphenol, it is reasonable to suggest that this compound may play a role in the therapeutic properties of rutin and of the extract of *P. peruviana* calyces. Thus, NCA was conducted for the quercetin data (representing the metabolites Q3OG and Q3OS). The PK parameters are detailed in Table 3.

The AUC_0-INF_ of quercetin (representing the conjugates) from pure rutin was proportional to the doses and Tmax was 6 h, but for quercetin from the extract, there was a deviation of PK linearity at 1000 mg/kg, with AUC_0-INF_/dose up to 1.8 times greater than that of the other extract doses. Meanwhile, Tmax ranged from 1.8 h at 500 mg/kg to 0.6 h at 1000 mg/kg, indicating that the matrix of the extract increases the rate of absorption of rutin conjugates in a dose-dependent manner (Table 3).

It was strongly evidenced in this study that the matrix of the extract did not improve the bioavailability of rutin but produced an increase up to 11.4 times in the bioavailability of the conjugates Q3OG and Q3OS, measured as the Frel of quercetin (Table 3). As discussed above, this effect could be relevant as the conjugates, especially Q3OG, may contribute to the pharmacological activity demonstrated for the extract [8,10].

The increase in the bioavailability of conjugate metabolites from the extract of calyces of *P. peruviana* is consistent with previous studies by our group on the intestinal permeability of pure rutin and rutin in the extract using the Caco-2 cell line model. In that study, we found the highest formation of Q3OG and Q3OS by Caco-2 cells and the lowest apical efflux of the compounds (i.e., less resistance to absorption) when we analyzed the extract. In addition, the rutin in the extract was approximately 10 times more soluble than the pure compound itself. Since rutin was more soluble, it could be more metabolized in the intestine, leading to the production of more conjugates, which, in turn, could be better absorbed [9]. Based on a report by Abou-Baker and Rady [49], extracts of *P. peruviana* calyces may contain a slight amount of quercitrin, another quercetin precursor. However, previous studies by our group aiming to characterize the bioactive compounds in the hydroalcoholic extract using NMR and MS spectroscopy have not identified that compound [11,32]. Nevertheless, if quercitrin was present, it would not be sufficient to explain the increase observed in the bioavailability or rate of absorption of quercetin conjugates when the extract was orally administrated to the animals.

Other authors have reported the effects of plant matrices on the bioavailability of rutin, Q3OG, and Q3OS. Lu et al. [29] suggested that the presence of non-flavonoid components in the extracts may be decisive for improving the bioavailability of rutin. More concretely, Tamura et al. [30] demonstrated that concomitant administration of rutin and pectin increases the bioavailability of Q3OG and Q3OS by altering the intestinal function and the metabolic activity of mice gut flora. The fruits of *P. peruviana* are rich in pectin [50]; Therefore, it is possible that the calyces also contain this polysaccharide.

In addition to the effects on PK linearity, Tmax and Frel, the matrix extract produced an important decrease (up to 91%) in Cl/F and k parameters, which were also expressed in the increase in the MRT and t_1/2_ up to 5 and 10 times that of the pure compound, respectively. Such effects may be a consequence of the greater bioavailability of the conjugates from the extract. However, considering that Q3OG and Q3OS could be metabolized in hepatocytes or excreted by the action of several transporters [51,52], the lowest rate of elimination of quercetin conjugates could also be associated with metabolism saturation or renal secretion of those conjugates. Moreover, one of the transporters that participates in drug excretion into the urine and bile is P-gp [53]. Previous results of our group suggested that some components in the extract of *P. peruviana* calyces may act as inhibitors of this transporter and may also lead to the decrease observed in Cl/F and k [9].

In contrast, it was observed that the parameter Vdz/F decreased by up to 69.7% due to the matrix extract but was proportional to its dose (Table 3). This could be explained by the increase in systemic exposure of Q3OG and Q3OS by the extract [48] and by the possible events of saturation or displacement of the metabolites from blood plasma proteins, as mentioned above for i.v. administered rutin.

Based on these results, the extract of *P. peruviana* calyces is a promising source to increase the bioavailability of the active metabolites of rutin and also a potential tool favoring the use of plant residues in pharmaceutical products.

### 3.4. Population Pharmacokinetic (PopPK) Modeling

To better describe the matrix effects of the extract on the PK of rutin and its metabolites, a popPK analysis was carried out based on the data collected experimentally. This approach involves a more complete analysis of the variability in drug concentrations among individuals in order to confirm whether an external factor, such as the mixture of compounds in the extract, alters the PK of the drugs [35]. This analysis is also used to estimate parameters related to the double-peak phenomenon, which cannot be obtained by NCA.

When building the independent models for rutin and quercetin (representing Q3OG and Q3OS) from the pure compound and extract, it was found that the course of drug plasma concentrations followed the same structural model for both treatments in each administration route. It was hypothesized that the matrix extract would influence the PK parameters but not the pharmacokinetic model of the compounds. Thus, two popPK models were developed: one to obtain the PK parameters of rutin after i.v. administration of the pure compound or the extract of *P. peruviana* calyces and the other to obtain the PK parameters of Q3OG and Q3OS measured as quercetin after oral administration of pure rutin or the extract. A summary of the main steps performed during the development of the model is presented in Tables S1 and S2.

**Figure 3:**
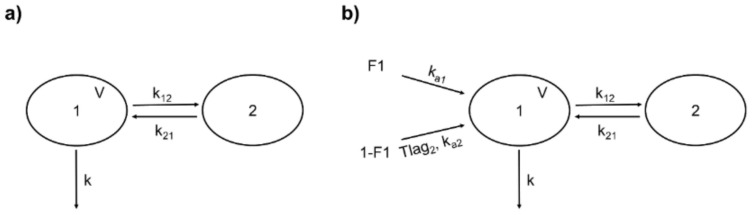
Structure of the population pharmacokinetic (popPK) models for rutin and quercetin conjugates (as quercetin). (**a**) Two-compartment model for intravenous rutin. 1: central compartment; 2: peripheral compartment; V: central compartment volume; k: first-order elimination rate constant; k_12_: rate constant of distribution from 1 to 2; k_21_: rate constant of distribution from 2 to 1. (**b**) Double absorption and two-compartment model for quercetin conjugates from oral rutin. F1: absorbed fraction from the first site; 1-F1: absorbed fraction from the second site; ka_1_: first-order absorption rate constant from the first site; ka_2_: first-order absorption rate constant from the second site; Tlag_2_: delay for the second absorption; other parameters are identical to (**a**).

Rutin (i.v.) from both sources was better fitted to a two-compartment model described by the following parameters: volume of the central compartment (V), *k*, and the distribution rate constants *k_12_* and *k_21_* (Figure 3a). The +99+986 differential equations representing this model are expressed in Equations (3) and (4), where *X_1_* and *X_2_* are the amounts of the drug in the central and peripheral compartments, respectively.

(3) $$\frac{dX_{1}}{dt}=k_{21}X_{2}-\left(k_{12}+k\right)X_{1}$$

(4) $$\frac{dX_{2}}{dt}=k_{12}X_{1}-k_{21}X_{2}$$

Based on the analysis of covariates, it was observed that the extract significantly influenced the V and k parameters but the doses were not a significant source of variability. Again, this shows the PK linearity of pure rutin and of the extract, as already observed in the NCA. Furthermore, a linear correlation was found between the random effects of V and k, indicating that k is dependent on V for individuals. The population parameters in Table 4 correspond to those of pure rutin (for the dosage set) that was used as a reference in the analysis of covariates. A similarity was noted between the values for *Vpop* (0.0646 L/kg) and Vdz of pure rutin from the NCA, especially at 2.9 mg/kg (0.068 L/kg). The Clpop parameter, estimated from *Vpop* and *kpop*, was equivalent to the Cl of pure rutin from the NCA (in both cases 0.095 L/h/kg for the set of doses). Table 4 also shows parameter β that represents the variability of PK parameters due to the significant covariates; the positive β values indicate that the matrix of the extract increases the *V* and *k* of rutin, as observed in the NCA. Equations (5)–(8) describe the model of covariates, where *η* is the individual deviation from Ppop.

(5) $$log(V/\left(1-V\right))=log(V_{pop}/\left(1-V_{pop}\right))+\beta_{V}GEXT+\eta_{V}$$

(6) $$\log\left(k\right)=\log\left(k_{pop}\right)+\beta_{k}GEXT+\eta_{k}$$

(7) $$\log\left(k_{12}\right)=\log\left(k_{12}{}_{pop}\right)$$

(8) $$\log\left(k_{21}\right)=\log\left(k_{21}{}_{pop}\right)$$

**Figure 4:**
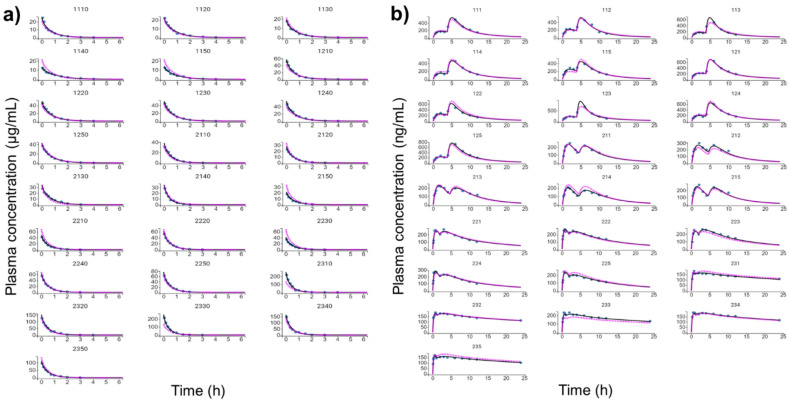
Individual and population-fitted plasma profiles of rutin and quercetin in rats using the nonlinear mixed-effect modeling approach (*n* = 5 for each dose). (**a**) Plasma profiles of rutin after intravenous administration of pure compound (RU) or hydroalcoholic extract from *Physalis peruviana* (HEE). 1110–1150: RU 1.45 mg/kg; 1210–1250: RU 2.9 mg/kg; 2110–2150: HEE 250 mg/kg (equivalent to 3.7 mg/kg of RU); 2210–2250: HEE 500 mg/kg (equivalent 7.4 mg/kg of RU); 2310–2350: HEE 1000 mg/kg (equivalent to 14.8 mg/kg of RU). (**b**) Plasma profiles of quercetin (representing the conjugates quercetin-3-*O*-glucuronide and quercetin-3-*O*-sulfate) after oral administration of RU or HEE. 111–115: RU 75 mg/kg; 121–125: RU 100 mg/kg; 211–215: HEE 500 mg/kg (equivalent to 7.4 mg/kg of RU); 221–225: HEE 750 mg/kg (equivalent to 11.1 mg/kg of RU); 231–235: HEE 1000 mg/kg (equivalent to 14.8 mg/kg of RU). Blue dots: observations; solid black line: individual pharmacokinetics model predictions; dashed violet line: population model predictions.

**Figure 5:**
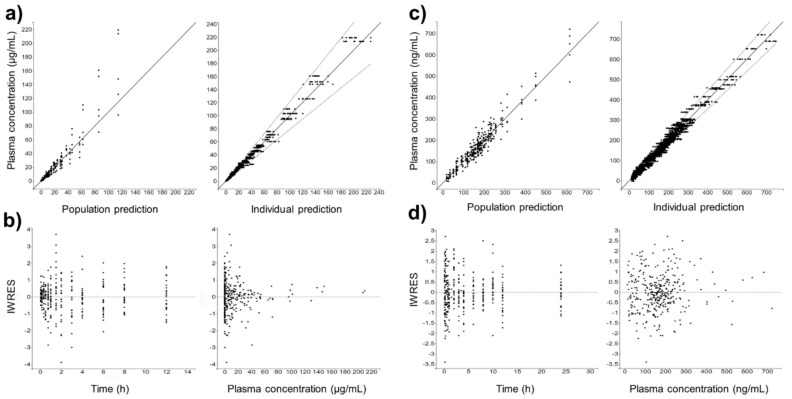
Goodness-of-fit for final popPK models. (**a**) Observation vs. prediction plots using population and individual parameters (including 10 predictions for each observation) for intravenous rutin; solid and dashed lines represent the linear regression fit and the 90% prediction interval, respectively. (**b**) Scatter plots of individual weighted residuals (IWRES) with respect to time and predicted plasma concentration for intravenous rutin. (**c**) Identical to (**a**) for quercetin from oral rutin. (**d**) Identical to (**b**) for quercetin from oral rutin. Quercetin represents the conjugates quercetin-3-*O*-glucuronide and quercetin-3-*O*-sulfate.

**Figure 6:**
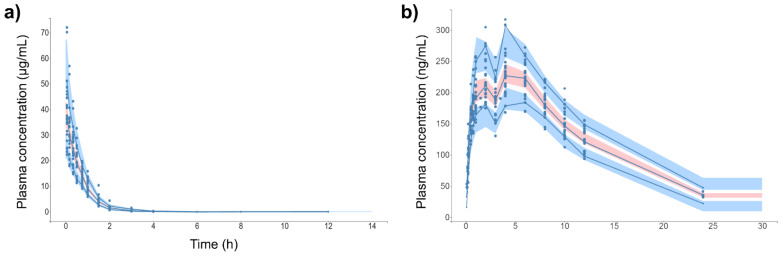
Visual predictive check (VPC) plot of final popPK models. (**a**) Intravenous rutin. (**b**) Quercetin from oral rutin. Blue dots are the observations (*n* = 25). Solid blue lines are the empirical 90th percentile. Blue and red shaded areas represent the 90% prediction interval based on 1000 simulated data. Quercetin represents the conjugates quercetin-3-*O*-glucuronide and quercetin-3-*O*-sulfate.

Figure 4 shows the individual and population-fitted blood plasma profiles of rutin for the complete model. The observations were better fitted to a combined residual error model (lowest AIC, 4121), and the residuals showed symmetrical distribution around zero (Figure 5b). The observation-versus-prediction plot also showed a good fit of the experimental data to the final model (Figure 5a), and the VPC plot suggested that the model adequately predicts the blood plasma concentrations of rutin (Figure 6a).

Quercetin (representing the conjugates Q3OG and Q3OS) derived from pure rutin or from the extract from the oral experiments also followed a two-compartment distribution model, but it was necessary to add new parameters to the model to describe the absorption process, since the plasma concentration curve of the drug exhibited two peaks (Figure 2c,d). According to the literature, the double-peak phenomenon of flavonoids may be due to the enteric recirculation of glucuronide conjugates that have been effluxed from the enterocytes in the small intestine or in the proximal region of the colon and reabsorbed after losing the conjugate by the action of glucuronidases from gut bacterial flora, i.e., there would be two absorption sites [16,17,54]. In the case of the glycosylated flavonoid, rutin, the second absorption in the colon would also be an advantage due to the abundance of glycosidases of the gut flora that would release the aglycone quercetin, which is more permeable than their glycosides [55].

Based on the above findings, we chose for quercetin (representing the conjugates Q3OG and Q3OS) two-site absorption (enteric recirculation) with two-compartment distribution and a first-order elimination model (Figure 3b). The differential equations of the model for central and peripherical compartments are expressed in Equations (9) and (10).

(9) $$\frac{dX_{1}}{dt}=k_{a1}F_{1}+\delta_{tTlag2}k_{a2}\left(1-F_{1}\right)+k_{21}X_{2}-\left(k_{12}+k\right)X_{1}$$

(10) $$\frac{dX_{2}}{dt}=k_{12}X_{1}-k_{21}X_{2},$$

where *X_1_* and *X_2_* are the amounts of quercetin in the central and peripheral compartments, respectively; ka_1_ is the rate constant of absorption from site 1; *F_1_* is the fraction of quercetin absorbed at site 1; ka_2_ is the rate constant of absorption from site 2; and is the fraction of quercetin absorbed at site 2. Since conceptually, the second absorption occurs with a delay from the first one equal to Tlag_2_, we included in Equation (9) the function *δ_tTlag2_*, which takes a value of 0 when the time is less than Tlag_2_ and 1 when the time is equal to or greater than Tlag_2_ (*δ_tTlag2_* = 0 if t < Tlag_2_ and *δ_tTlag2_* = 1 if t ≥ *Tlag_2_*).

According to the deviation from linear PK observed in the NCA, the different doses of pure rutin and the extract were significant categorical covariates in the model, and the parameters affected by them were ka_1_, *Tlag_2_*, *V*, and *k*. The dose of 100 mg/kg of pure rutin was used as a reference, and its population parameters are detailed in Table 5, including the β coefficient for the significant covariates. Equations (11)–(18) describe the model of covariates, with individual deviation from Ppop, *η*.

(11) $$\log\left(ka_{1}\right)=\log\left(ka_{1_{pop}}\right)+\beta_{ka1}D75+\beta_{ka1}D500+\beta_{ka1}D750+\beta_{ka1}D1000+\eta_{ka1}$$

(12) $$\log\left(ka_{2}\right)=\log\left(Ka_{2\_pop}\right)+\eta_{ka2}$$

(13) $$log(F_{1}/\left(1-F_{1}\right))=log(F_{1\_pop}/\left(1-F_{1\_pop}\right))+\eta_{F1}$$

(14) $$\log\left(Tlag_{2}\right)=\log\left(Tlag_{2\_pop}\right)+\beta_{Tlag2}D75+\beta_{Tlag2}D500+\beta_{Tlag2}D750+\beta_{Tlag2}D1000+\eta_{Tlag2}$$

(15) $$\log\left(V\right)=\log\left(V_{pop}\right)+\beta_{V}D75+\beta_{V}D500+\beta_{V}D750+\beta_{V}D1000+\eta_{V}$$

(16) $$\log\left(k\right)=\log\left(k_{pop}\right)+\beta_{k}D75+\beta_{k}D500+\beta_{k}D750+\beta_{k}D1000+\eta_{k}$$

(17) $$\log\left(k_{12}\right)=\log\left(k_{12}{}_{pop}\right)$$

(18) $$\log\left(k_{21}\right)=\log\left(k_{21}{}_{pop}\right)$$

As shown in Table 5, the *β_ka1_* values increased proportionally with the extract dose, indicating that the higher the extract dose, the greater the rate of the first absorption of quercetin conjugates. Consequently, the *Tlag_2_* decrease is inverse to the extract dose, and these effects were also responsible for the decrease in Tmax observed in the NCA (Table 3). The model also showed that the V parameter for the orally administrated experiments starts off lower than that of the pure compound (negative *β_V_* at 500 mg/kg) but increases with the extract dose (positive *β_V_* at 750 and 1000 mg/kg). In contrast, NCA showed that the *k* parameter decreases when the extract dose increases, and this dose dependency was determined in the model by the parameters *β_k_* for each dose that presented negative values. The probable explanations for all these changes are discussed above.

Figure 4b shows the individual and population-fitted blood plasma profiles of quercetin (representing the conjugates Q3OG and Q3OS) for the complete model. The model was able to correctly describe the double-peak phenomenon and the effects of the extract matrix on the PK of quercetin conjugates in rats, and these effects were also dose dependent. In this case, the residual error was also described by a combined model (lowest AIC, 2648), and the plots of residuals and observation versus prediction (Figure 5c,d) confirmed that the final model was appropriate for describing the experimental data. The VPC plot was also adequate for internal validation of the model (Figure 6b).

Based on our results, popPK analysis confirmed the effects of the extract matrix on the distribution and elimination of rutin after i.v. administration and on the absorption rate, distribution, and elimination of Q3OG and Q3OS from rutin, after oral administration, using primary data and only one software program.

## 4. Conclusions

The matrix of the extract of calyces of *P. peruviana* significantly increases the volume of distribution and rutin clearance, as well as the rate of absorption and oral bioavailability of Q3OG and Q3OS measured as quercetin. The popPK models developed (two compartments with linear elimination and double absorption) correctly describe the differences between rutin from the extract and pure rutin, including the dose dependency. These effects should be considered for the rational design of botanical preparations with this extract.

## Figures and Tables

**Table 1:** Groups for pharmacokinetic (PK) studies.

Group	Intervention	Administration Route	Dose of Intervention (mg/kg)
1	Pure rutin	i.v.	1.45
2			2.9
3		p.o.	75
4			100
5	Extract of calyces of *P. peruviana*	i.v.	250/3.7 *
6			500/7.4 *
7			1000/14.8 *
8		p.o.	500/7.4 *
9			750/11.1 *
10			1000/14.8 *

* The dose is expressed as rutin equivalent according to the content of this flavonoid in the extract (14.8 µg/kg).

**Table 2:** Pharmacokinetic parameters from non-compartmental analysis after intravenous administration of pure rutin and extract of calyces of *Physalis peruviana*.

Parameter	Rutin		*P. peruviana* Extract		
	Dose (mg/kg)				
	1.45	2.9	250	500	1000
AUC_0-INF_ (ng*h/mL)	14,853.11 ± 2528.037	33,933.58 ± 2878.316	16,932.91 ± 4568.149	28,821.05 ± 6633.516	80,696.50 ± 19,536.208
Vdss (L/kg)	0.11 ± 0.0306	0.07 ± 0.009	0.18 ± 0.056 #	0.18 ± 0.052 #	0.11 ± 0.040
Cl (L/h/kg)	0.10 ± 0.017	0.09 ± 0.008	0.23 ± 0.065 *#	0.27 ± 0.067 *#	0.19 ± 0.049 *#
k (h^−1^)	1.08 ± 0.195	1.29 ± 0.159	1.67 ± 0.312 *	1.77 ± 0.05 *#	1.96 ± 0.175 *#
t_1/2_ (h)	0.66 ± 0.130	0.54 ± 0.062	0.43 ± 0.070 *	0.39 ± 0.011 *#	0.36 ± 0.031 *##
MRT (h)	1.08 ± 0.176	0.81 ± 0.053 *	0.78 ± 0.089 *	0.66 ± 0.027 *	0.54 ± 0.078 *# ¥
Vdz (L/kg)	0.096 ± 0.027	0.068 ± 0.012	0.14 ± 0.039 #	0.15 ± 0.043 #	0.099 ± 0.029
AUC/dose × 10^−3^ (h/L)	10,243.52 ± 1743.478	11,701.24 ± 992.522	4576.46 ± 1234.634 *#	3894.74 ± 896.421 *#	5452.47 ± 1320.012 *#

Data are expressed as the mean ± standard deviation of *n* = 5. AUC_0-INF_: area under the curve to infinity; Vdss: volume of distribution at the steady state; Cl: clearance; k: first-order rate constant of elimination; t_1/2_: terminal half-life; MRT: mean residence time; Vdz: volume of distribution associated with the terminal phase; AUC/dose: ratio of the AUC_0-INF_ to the dose. * Significantly different from pure rutin 1.45 mg/kg (*p* < 0.05); # significantly different from pure rutin 2.9 mg/kg (*p* < 0.05); ## significantly different from pure rutin 2.9 mg/kg (*p* < 0.01); ¥ significantly different from extract 250 mg/kg (*p* < 0.05). ANOVA and HSD/Mann–Whitney.

**Table 3:** Pharmacokinetic parameters from non-compartmental analysis of quercetin after oral administration of pure rutin and the extract of calyces of *Physalis peruviana*.

Parameter	Rutin		Extract		
	Dose (mg/kg)				
	75	100	500	750	1000
AUC_0-INF_ (ng*h/mL)	4095.34 ± 227.038	5227.34 ± 469.187	3197.85 ± 198.808	3724.53 ± 274.346	9074.85 ± 737.580
Cmax (ng/mL)	462.78 ± 49.310	627.51 ± 96.807	254.55 ± 39.385	266.72 ± 11.914	193.41 ± 25.174
Tmax (h)	6	6	1.80 ± 0.274 *#	1.60 ± 1.282 *#	0.60 ± 0.137 *# ¥
Vdz/F (L/kg)	86.08 ± 12.200	87.12 ± 13.980	26.40 ± 3.714 *#	35.76 ± 4.681 *#	79.98 ± 10.112 *# ¥ ƶ
Cl/F (L/h/kg)	18.36 ± 0.963	19.26 ± 1.699	2.32 ± 0.144 *#	2.99 ± 0.214 *#	1.64 ± 0.125 *#
k (h^−1^)	0.22 ± 0.034	0.22 ± 0.033	0.09 ± 0.010 *#	0.08 ± 0.005 *#	0.02 ± 0.002 ¥ ƶ
t_1/2_ (h)	3.25 ± 0.446	3.13 ± 0.391	7.87 ± 0.907 *#	8.26 ± 0.545 *#	33.82 ± 3.412 ¥ ƶ
MRT (h)	9.29 ± 0.323	8.98 ± 0.316	12.38 ± 1.211	12.11 ± 0.633	49.06 ± 4.864 *# ¥ ƶ
AUC/dose × 10^−3^ (h/L)	54.61 ± 3.026	52.27 ± 4.691	432.14 ± 26.864 *#	335.54 ± 24.714 *# ¥	613.16 ± 49.838 *# ¥ ƶ
Frel	1.0	1.0	7.9	6.1	11.2

Quercetin represents the metabolites quercetin-3-*O*-glucuronide and quercetin-3-*O*-sulfate. Data are expressed as the mean ± standard deviation of *n* = 5. AUC_0-INF_: area under the curve to infinity; Cmax: maximum observed concentration; Tmax: time of maximum observed concentration; Vdz/F: volume of distribution associated with the terminal phase over bioavailability; Cl/F: clearance over bioavailability; k: first-order rate constant of elimination; t_1/2_: terminal half-life; MRT: mean residence time; AUC/dose: ratio of the AUC_0-INF_ to the dose; Frel: relative bioavailability. * Significantly different from pure rutin 75 mg/kg (*p* < 0.05); # significantly different from pure rutin 100 mg/kg (*p* < 0.05); ¥ significantly different from extract 500 mg/kg (*p* < 0.05); ƶ significantly different from extract 750 mg/kg (*p* < 0.05). ANOVA and HSD/Mann–Whitney.

**Table 4:** Population pharmacokinetic parameters of rutin after intravenous administration of pure rutin and from the extract from *Physalis peruviana* calyces.

	Value	Stochastic Approximation	
		S.E.	R.S.E. (%)
**Fixed Effects**			
V_pop_ (L/kg)	0.0646	0.00662	10.2
β_V_GEXT	0.478	0.128	26.8
k_pop_ (h^−1^)	1.47	0.0812	5.54
β_k_GEXT	0.395	0.0622	15.8
k_12_pop_ (h^−1^)	2.6	1.1	42.3
k_21_pop_ (h^−1^)	13.6	4.37	32.2
**Standard Deviation of the Random Effects**			
Ω V	0.306	0.0469	15.3
Ω k	0.147	0.0231	15.8
**Correlations**			
k V	−0.701	0.111	15.8
**Error Model Parameters**			
a	38.9	6.47	16.6
b	0.128	0.00801	6.28

Pop: population parameter; S.E.: standard error; R.S.E.: relative standard error; V: volume of the central compartment; k: first-order rate constant of elimination; k_12_: rate constant of drug distribution from the central to the peripherical compartment; k_21_: rate constant of drug distribution from the peripherical to the central compartment; β: variability due to significant covariate; GEXT: covariate extract; Ω: random variability; a and b: residual error components.

**Table 5:** Population pharmacokinetic parameters of quercetin after oral administration of pure rutin and of the extract of calyces of *Physalis peruviana*.

	Value	Stochastic Approximation	
		S.E.	R.S.E. (%)
**Fixed Effects**			
ka_1_pop_ (h^−1^)	0.104	0.0129	12.5
β_ka1_D75	0.281	0.167	59.6
β_ka1_D500	1.94	0.166	8.54
β_ka1_D750	3.15	0.176	5.59
β_ka1_D1000	3.77	0.175	4.64
ka_2_pop_ (h^−1^)	0.411	0.0686	16.7
F_1_pop_	0.565	0.0275	4.87
Tlag_2_pop_ (h)	3.91	0.021	0.538
β_Tlag2_D75	−0.0177	0.00556	31.3
β_Tlag2_D500	0.149	0.0936	63
β_Tlag2_D750	−0.695	0.0595	8.56
β_Tlag2_D1000	−1.17	0.254	21.7
V_pop_ (L/kg)	13.2	1.14	8.68
β_V_D75	0.251	0.0951	37.8
β_V_D500	−0.645	0.105	16.4
β_V_D750	0.245	0.118	48.2
β_V_D1000	1.09	0.101	9.25
k_pop_ (h^−1^)	1.42	0.119	8.35
β_k_D75	−0.225	0.0824	36.6
β_k_D500	−1.3	0.144	11.2
β_k_D750	−2.13	0.181	8.49
β_k_D1000	−3.57	0.231	6.47
k_12_pop_ (h^−1^)	0.279	0.0802	28.8
k_21_pop_ (h^−1^)	0.268	0.107	39.9
**Standard Deviation of the Random Effects**			
Ω ka_1_	0.189	0.0429	22.7
Ω ka_2_	0.236	0.0559	23.8
Ω F_1_	0.214	0.0769	36
Ω V	0.0819	0.0148	18.1
**Error Model Parameters**			
a	8.87	2.49	28.1
b	0.0457	0.0143	31.3

Quercetin represents the metabolites quercetin-3-*O*-glucuronide and quercetin-3-*O*-sulfate. Pop: population parameter; S.E.: standard error; R.S.E.: relative standard error; ka1: first-order absorption rate constant from the first site; ka2: first-order absorption rate constant from the second site; F1: absorbed fraction from the first site; Tlag2: delay for the second absorption; V: volume of the central compartment; k: first-order rate constant of elimination; k12: rate constant of drug distribution from the central to the peripherical compartment; k21: rate constant of drug distribution from the peripherical to the central compartment; β: variability due to significant covariate; D75: covariate pure rutin dose 75 mg/kg; D500, D750, D1000: covariate extract doses 500, 750, and 1000 mg/kg, respectively; Ω: random variability; a and b: residual error components.

## Data Availability

Publicly available datasets were analyzed in this study. This data can be found here: https://repositorio.unal.edu.co/handle/unal/78924 (accessed on 1 March 2021).

## Supplementary Materials

The following are available online at https://www.mdpi.com/article/10.3390/pharmaceutics13040535/s1, Figure S1: Chromatographic profile of the hydroethanolic extract of calyces of *Physalis peruviana*, Figure S2: Selectivity and specificity of the bioanalytical method, Table S1: Summary of run record for modeling of rutin in intravenously administration experiments, Table S2: Summary of run record for modeling of quercetin in oral administration experiments.
