# Supplementary material for: Matrix Effects of the Hydroethanolic Extract of Calyces of Physalis peruviana L. on Rutin Pharmacokinetics in Wistar Rats Using Population Modeling
Source: Pharmaceutics. 2021 Apr 12;13(4):535. doi: 10.3390/pharmaceutics13040535 (PMC8069016; doi:10.3390/pharmaceutics13040535)
Supplement: Supplementary file 1 [file pharmaceutics-13-00535-s001.pdf]

pharmaceutics

Supplementary Materials: Matrix Effects of the Hydroethanolic Extract of Calyces of *Physalis peruviana* L. on Rutin Pharmacokinetics in Wistar Rats Using Population Modeling

Gina Paola Domínguez Moré, María Isabel Cardona, Paula Michelle Sepúlveda, Sandra Milena Echeverry, Cláudia Maria Oliveira Simões and Diana Marcela Aragón

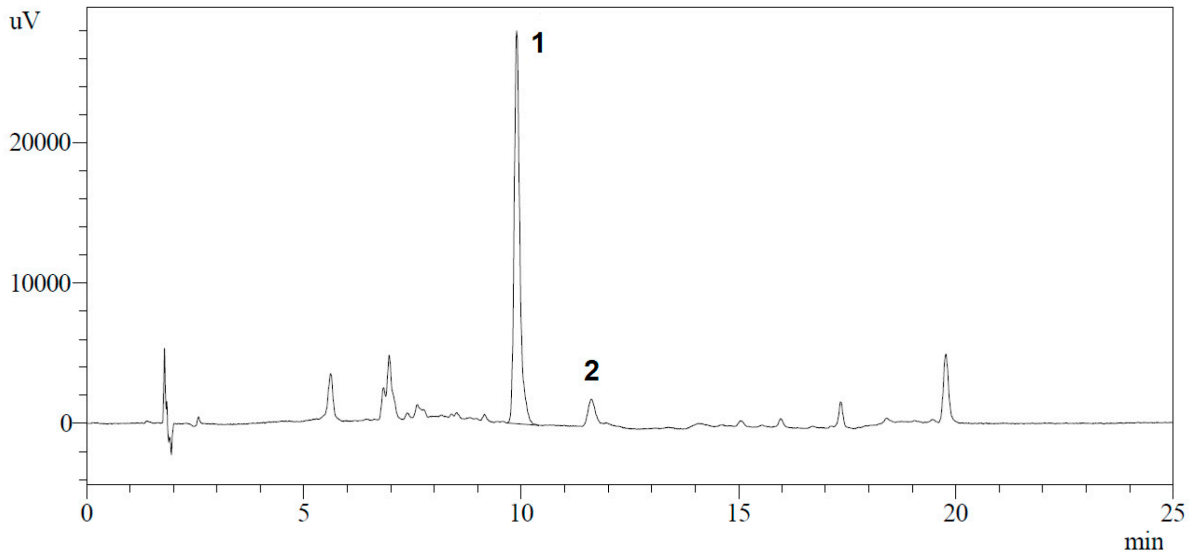

Figure S1. Chromatographic profile of the hydroethanolic extract of calyces from *Physalis peruviana.* **1:** rutin, retention time 9.8 min. **2:** Nicotiflorin, retention time 10.5 min. Column C18 (150 × 3.9 mm, 10 µm), mobile phase with methanol (A) and water, acidified with acetic acid at 0.5%, in gradient from 10 to 80% A, over 25 min at 1 mL/min; column oven at 35ºC, detection at 350 nm and injections of 10 µL of the samples diluted in methanol (1 mg/mL) [39].

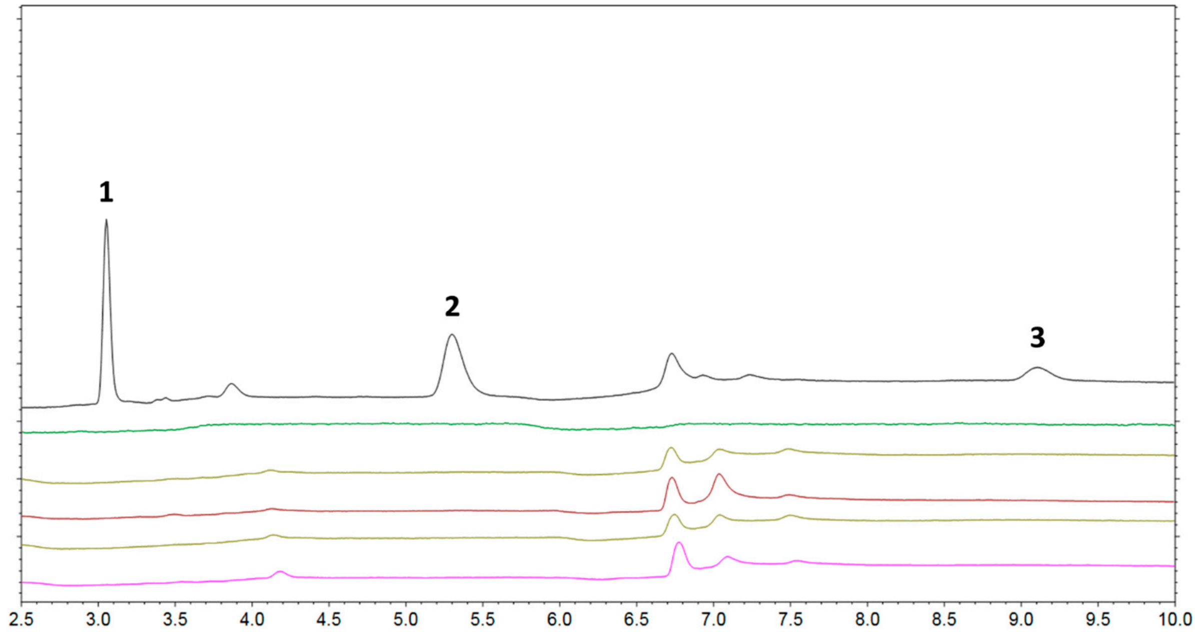

Figure S2. Selectivity and specificity of the bioanalytical method. Colored lines are blank samples from different rats. Black line is blank plasma spiked with rutin (peak 1, 3.1 min) and quercetin (peak 2, 5.4 min) at concentration of 250 ng/mL, the internal standard (IS) chrysin (peak 3, 9.1 min) at concentration of 1000 ng/mL and quercetin-3-*O*-glucuronide for potential interference evaluation. There was not interfering signals for the analytes and IS. Column Kinetex® EVO C18, 2,6 µm, 100 × 2.1 mm at 30ºC, mobile phase with formic acid 0.1% (A) and acetonitrile acidified with formic acid at 0.5%, in gradient from 85 to 65% A, at 0.5 mL/min. Detection at 260 nm and injections of 6 µL of the samples. Samples were prepared by protein precipitation with methanol (1:2).

Table S1. Summary of run record for modelling of rutin in intravenously administration experiments.

Run Description	Results			
Pure rutin: one compartment model, first order elimination.	**Parameter**	**Value**	**SE**	**RSE (%)**
	**V_pop**	$7.81 e^{-5}$	$6.51 e^{-6}$	8.33
	**k_pop**	1.22	0.0604	4.97
	AIC: 1656			
Pure rutin: two-compartment model, first order elimination.	**Parameter**	**Value**	**SE**	**RSE (%)**
	**V_pop**	$5.89e^{-5}$	$2.85 e^{-6}$	4.83
	**k_pop**	1.61	0.00181	0.113
	**k12_pop**	5.24	1.33	25.4
	**k21_pop**	19.4	0.151	0.779
	AIC: 1646			
Pure rutin: selection of model error and final model	**Parameter**	**Value**	**SE**	**RSE (%)**
	**V_pop**	$6.4e^{-5}$	$3.37 e^{-6}$	5.27
	**k_pop**	1.48	0.04	2.69
	**k12_pop**	2.5	1.77	8.35
	**k21_pop**	16.8	11.7	4.96
	AIC: 1644			
Extract: one compartment model, first order elimination.	**Parameter**	**Value**	**SE**	**RSE (%)**
	**V_pop**	0.000128	$1.08 e^{-5}$	8.48
	**k_pop**	1.8	0.0623	3.45
	AIC: 2478			
Extract: two-compartment model, first order elimination and final model.	**Parameter**	**Value**	**SE**	**RSE (%)**
	**V_pop**	0.000106	$8.49 e^{-6}$	8.04
	**k_pop**	2.18	0.0722	3.31
	**k12_pop**	2.3	0.0622	20.6
	**k21_pop**	13	0.00193	0.00935
	AIC: 2470			
Pure rutin and extract model: model of covariate 1.	**Parameter**	**Value**	**SE**	**RSE (%)**
	**V_pop**	$5.9 e^{-5}$	$1.12 e^{-5}$	19.1
	**beta_V_tCOVARIATE_2_G_EXT**	0.458	0.126	27.5
	**k_pop**	1.58	0.258	16.3
	**beta_k_tCOVARIATE_2_G_EXT**	0.404	0.0587	14.5
	**k12_pop**	2.62	2.73	**104**
	**k21_pop**	9.67	3.6	37.2
	AIC: 4197			
Pure rutin and extract model: final model.	**Parameter**	**Value**	**SE**	**RSE (%)**
	**V_pop**	$6.46 e^{-5}$	$6.62 e^{-6}$	10.2
	**beta_V_tCOVARIATE_2_G_EXT**	0.478	0.128	26.8
	**k_pop**	1.47	0.0812	5.54
	**beta_k_tCOVARIATE_2_G_EXT**	0.395	0.0622	15.8
	**k12_pop**	2.6	1.1	42.3
	**k21_pop**	13.6	4.37	32.2
	AIC: 4121			

Pop: population parameter. S.E.: standard error. R.S.E.: relative standard error. V: volume of the central compartment, k: first order rate constant of elimination, k12 rate constant of drug distribution from central to the peripherical compartment, k21 rate constant of drug distribution from peripherical to central compartment, β: variability due to significant covariate, GEXT: covariate extract.

Table S2. Summary of run record for modelling of quercetin in oral administration experiments.

Run Description	Results			
Pure rutin: one compartment model, two first order absorption, first order elimination.	**Parameter**	**Value**	**SE**	**RSE (%)**
	**ka1_pop**	0.992	0.0676	6.81
	**ka2_pop**	3.33	1.67	**50.2**
	**F1_pop**	0.336	0.0229	6.79
	**Tlag2_pop**	3.93	0.0351	0.892
	**V_pop**	0.0868	0.00204	2.35
	**k_pop**	0.241	0.00989	4.1
	AIC: 1067			
Pure rutin: two-compartment model, two first order absorption, first order elimination.	**Parameter**	**Value**	**SE**	**RSE (%)**
	**ka1_pop**	0.605	0.107	17.6
	**ka2_pop**	1.1	0.241	21.9
	**F1_pop**	0.358	0.0268	7.49
	**Tlag2_pop**	3.85	0.0273	0.707
	**V_pop**	0.0566	0.00865	15.3
	**k_pop**	0.344	0.0581	16.9
	**k12_pop**	0.232	0.0912	39.2
	**k21_pop**	0.336	0.137	40.8
	AIC: 1047			
Pure rutin: evaluation of random effects.	**Parameter**	**Value**	**SE**	**RSE (%)**
	**ka1_pop**	0.614	0.0373	6.07
	**ka2_pop**	2.35	0.502	21.4
	**F1_pop**	0.36	0.0257	7.15
	**Tlag2_pop**	3.94	0.0104	0.265
	**V_pop**	0.0562	0.00262	4.66
	**k_pop**	0.352	0.0192	5.45
	**k12_pop**	0.324	0.0808	25
	**k21_pop**	0.659	0.143	21.6
	AIC: 1037			
Pure rutin: selection of model error and final model	**Parameter**	**Value**	**SE**	**RSE (%)**
	**ka1_pop**	0.277	0.136	49
	**ka2_pop**	0.545	0.145	26.7
	**F1_pop**	0.411	0.0684	16.7
	**Tlag2_pop**	3.86	0.0312	0.809
	**V_pop**	0.0284	0.0115	40.4
	**k_pop**	0.707	0.29	41
	**k12_pop**	0.391	0.0908	23.2
	**k21_pop**	0.29	0.132	45.5
	AIC: 1034			
Extract: one compartment model, two first order absorption, first order elimination.	Convergence did not reach.			
Extract: two-compartment model, two first order absorption, first order elimination.	**Parameter**	**Value**	**SE**	**RSE (%)**
	**ka1_pop**	1.28	0.176	13.8
	**ka2_pop**	0.485	0.133	27.4
	**F1_pop**	0.62	0.0494	7.96
	**Tlag2_pop**	2.3	0.371	16.1
	**V_pop**	0.0114	0.000797	7

Run Description	Results			
	**k_pop**	0.224	0.0216	9.65
	**k12_pop**	0.776	0.349	45
	**k21_pop**	0.543	0.0248	4.56
	AIC: 1699			
Extract: initial selection of covariates	**Parameter**	**Value**	**SE**	**RSE (%)**
	**ka1_pop**	0.803	0.159	19.8
	**beta_ka1_COVARIATE_D2**	1.08	0.203	18.8
	**beta_ka1_COVARIATE_D3**	1.8	0.219	12.2
	**ka2_pop**	0.469	0.144	30.8
	**F1_pop**	0.616	0.0456	7.4
	**Tlag2_pop**	4.46	0.601	13.5
	**beta_Tlag2_COVARIATE_D2**	−0.799	0.162	20.3
	**beta_Tlag2_COVARIATE_D3**	−1.78	0.228	12.8
	**V_pop**	0.00814	0.0017	20.9
	**beta_V_COVARIATE_D2**	0.782	0.153	19.5
	**beta_V_COVARIATE_D3**	1.65	0.16	9.67
	**k_pop**	0.328	0.0801	24.4
	**beta_k_COVARIATE_D2**	−0.69	0.197	28.6
	**beta_k_COVARIATE_D3**	−2.2	0.247	11.2
	**k12_pop**	0.308	0.0816	26.5
	**k21_pop**	0.347	0.104	30.1
	AIC: 1575			
Extract: evaluation of random effects.	**Parameter**	**Value**	**SE**	**RSE (%)**
	**ka1_pop**	0.837	0.146	17.4
	**beta_ka1_COVARIATE_D2**	1.03	0.182	17.7
	**beta_ka1_COVARIATE_D3**	1.64	0.193	11.8
	**ka2_pop**	0.495	0.123	24.9
	**F1_pop**	0.615	0.0398	6.48
	**Tlag2_pop**	4.25	0.701	16.5
	**beta_Tlag2_COVARIATE_D2**	−0.756	0.174	23.1
	**beta_Tlag2_COVARIATE_D3**	−1.16	0.228	19.7
	**V_pop**	0.0085	0.0015	17.6
	**beta_V_COVARIATE_D2**	0.737	0.131	17.9
	**beta_V_COVARIATE_D3**	1.55	0.138	8.91
	**k_pop**	0.297	0.0674	22.7
	**beta_k_COVARIATE_D2**	−0.665	0.187	28.1
	**beta_k_COVARIATE_D3**	−2.06	0.243	11.8
	**k12_pop**	0.314	0.0747	23.8
	**k21_pop**	0.301	0.0927	30.8
	AIC: 1566			
Extract: selection of model error and final model	**Parameter**	**Value**	**SE**	**RSE (%)**
	**ka1_pop**	0.833	0.109	13.1
	**beta_ka1_COVARIATE_D2**	0.974	0.186	19.1
	**beta_ka1_COVARIATE_D3**	1.63	0.178	11
	**ka2_pop**	0.487	0.105	21.5
	**F1_pop**	0.617	0.033	5.35
	**Tlag2_pop**	4.15	0.699	16.8
	**beta_Tlag2_COVARIATE_D2**	−0.73	0.168	23
	**beta_Tlag2_COVARIATE_D3**	−1.18	0.24	20.3
	**V_pop**	0.00837	0.000866	10.4
	**beta_V_COVARIATE_D2**	0.71	0.0991	14
	**beta_V_COVARIATE_D3**	1.55	0.0984	6.35
	**k_pop**	0.301	0.0403	13.4
	**beta_k_COVARIATE_D2**	−0.604	0.161	26.7
	**beta_k_COVARIATE_D3**	−2.04	0.188	9.21
	**k12_pop**	0.343	0.0862	25.1
	**k21_pop**	0.325	0.0937	28.8

Run Description	Results			
		0		
	AIC: 1549			
Pure rutin and extract model: model of covariate 1.	**Parameter**	**Value**	**SE**	**RSE (%)**
	**ka1_pop**	0.133	0.0191	14.3
	**beta_ka1_tCOVARIATE_1_G_D3**	−0.103	0.189	**184**
	**beta_ka1_tCOVARIATE_1_G_D4**	1.75	0.158	9.02
	**beta_ka1_tCOVARIATE_1_G_D5**	2.91	0.16	5.49
	**beta_ka1_tCOVARIATE_1_G_D6**	3.51	0.19	5.41
	**ka2_pop**	0.409	0.121	29.6
	**F1_pop**	0.559	0.0584	10.4
	**Tlag2_pop**	3.85	0.0489	1.27
	**beta_Tlag2_tCOVARIATE_1_G_D3**	0.0144	0.00591	40.9
	**beta_Tlag2_tCOVARIATE_1_G_D4**	0.105	0.11	**105**
	**beta_Tlag2_tCOVARIATE_1_G_D5**	−0.687	0.0613	8.92
	**beta_Tlag2_tCOVARIATE_1_G_D6**	−1.14	0.282	24.6
	**V_pop**	0.0153	0.00161	10.5
	**beta_V_tCOVARIATE_1_G_D3**	0.00836	0.147	**1.76 e^{+3}**
	**beta_V_tCOVARIATE_1_G_D4**	−0.737	0.119	16.2
	**beta_V_tCOVARIATE_1_G_D5**	0.0888	0.124	**140**
	**beta_V_tCOVARIATE_1_G_D6**	0.922	0.12	13
	**k_pop**	1.25	0.107	8.53
	**beta_k_tCOVARIATE_1_G_D3**	−5.64 e^{-5}	0.112	**1.99 e^{+5}**
	**beta_k_tCOVARIATE_1_G_D4**	−1.26	0.106	8.37
	**beta_k_tCOVARIATE_1_G_D5**	−2.01	0.123	6.13
	**beta_k_tCOVARIATE_1_G_D6**	−3.41	0.317	9.31
	**k12_pop**	0.289	0.171	**59.1**
	**k21_pop**	0.272	0.202	**74.2**
	AIC: 2652			
Pure rutin and extract model: final model.	**Parameter**	**Value**	**SE**	**RSE (%)**
	**ka1_pop**	0.104	0.0129	12.5
	**beta_ka1_tCOVARIATE_1_G_D2**	0.281	0.167	**59.6**
	**beta_ka1_tCOVARIATE_1_G_D4**	1.94	0.166	8.54
	**beta_ka1_tCOVARIATE_1_G_D5**	3.15	0.176	5.59
	**beta_ka1_tCOVARIATE_1_G_D6**	3.77	0.175	4.64
	**ka2_pop**	0.411	0.0686	16.7
	**F1_pop**	0.565	0.0275	4.87
	**Tlag2_pop**	3.91	0.021	0.538

Run Description	Results			
	**beta_Tlag2_tCOVARIATE_1_G_D2**	−0.0177	0.00556	31.3
	**beta_Tlag2_tCOVARIATE_1_G_D4**	0.149	0.0936	**63**
	**beta_Tlag2_tCOVARIATE_1_G_D5**	−0.695	0.0595	8.56
	**beta_Tlag2_tCOVARIATE_1_G_D6**	−1.17	0.254	21.7
	**V_pop**	0.0132	0.00114	8.68
	**beta_V_tCOVARIATE_1_G_D2**	0.251	0.0951	37.8
	**beta_V_tCOVARIATE_1_G_D4**	−0.645	0.105	16.4
	**beta_V_tCOVARIATE_1_G_D5**	0.245	0.118	48.2
	**beta_V_tCOVARIATE_1_G_D6**	1.09	0.101	9.25
	**k_pop**	1.42	0.119	8.35
	**beta_k_tCOVARIATE_1_G_D2**	−0.225	0.0824	36.6
	**beta_k_tCOVARIATE_1_G_D4**	−1.3	0.144	11.2
	**beta_k_tCOVARIATE_1_G_D5**	−2.13	0.181	8.49
	**beta_k_tCOVARIATE_1_G_D6**	−3.57	0.231	6.47
	**k12_pop**	0.279	0.0802	28.8
	**k21_pop**	0.268	0.107	39.9
	AIC: 2648			

Pop: population parameter. S.E.: standard error. R.S.E.: relative standard error. ka1: first order absorption rate constant from first site, ka2: first order absorption rate constant from second site, F1: absorbed fraction from first site, Tlag2: delay for second absorption, V: volume of the central compartment, k: first order rate constant of elimination, k12 rate constant of drug distribution from central to the peripherical compartment, k21 rate constants of drug distribution from peripherical to central compartment, β: variability due to significant covariate, G_D2: covariate pure rutin dose 75 mg/kg; G_D4, G_D5, G_6: covariate extract doses 500, 750 and 1000 mg/kg respectively.
